# Supplementary material for: β-Elemene derivatives produced from SeO2-mediated oxidation reaction
Source: R Soc Open Sci. 2020 May 13;7(5):200038. doi: 10.1098/rsos.200038 (PMC7277271; doi:10.1098/rsos.200038)

β-Elemene derivatives produced from SeO_2-mediated oxidation reaction

Xingrui He[1,2,3,4#], Xiao-Tao Zhuo[1,2,3,#], Yuan Gao[5], Renren Bai[6], Xiang-Yang Ye*[,1,2,3,7], Tian Xie*[,1,2,3,7]

ORCID ID's of all contributors:

Xingrui He: 0000-0002-7591-8022; Xiao-Tao Zhuo: 0000-0002-4388-4809; Yuan Gao: 0000-0003-2254-6381; Renren Bai: 0000-0002-3511-5794; Xiang-Yang Ye：0000-0003-3739-0930; Tian Xie：0000-0001-7066-1443

[1]Key Laboratory of Elemene Class Anti-Cancer Chinese Medicine of Zhejiang Province, [2]Engineering Laboratory of Development and Application of Traditional Chinese Medicine from Zhejiang Province, [3]Holistic Integrative Pharmacy Institutes (HIPI), School of Medicine, Hangzhou Normal University, Hangzhou, Zhejiang 311121, P.R.China. [4]School of Pharmacy, Liaocheng University, Shandong 252000, P.R.China. [5]School of Clinical Medicine, Guangdong Pharmaceutical University, Guangzhou, Guangdong 510000, P.R.China. [6]College of Pharmaceutical Science, Zhejiang University of Technology, Hangzhou, Zhejiang 310014, P.R.China. [7]Collaborative Innovation Center of Chinese Medicines from Zhejiang Province, Hangzhou Normal University, Hangzhou, Zhejiang 311121, P.R.China.

**Address correspondence to these authors at the Holistic Integrative Pharmacy Institutes (HIPI), School of Medicine, Hangzhou Normal University, Hangzhou, Zhejiang 311121, P.R.China;*

[#]These two authors contributed equally

Tel: +86-571-28860236 (Xiang-Yang Ye); E-mail: xyye@hznu.edu.cn (Xiang-Yang Ye);

Tel: +86-571-28860237 (Tian Xie); E-mail: xbs@hznu.edu.cn (Tian Xie);

Supporting Information

^{1}H NMR (500 MHz, Chloroform-*d*) data of **3**

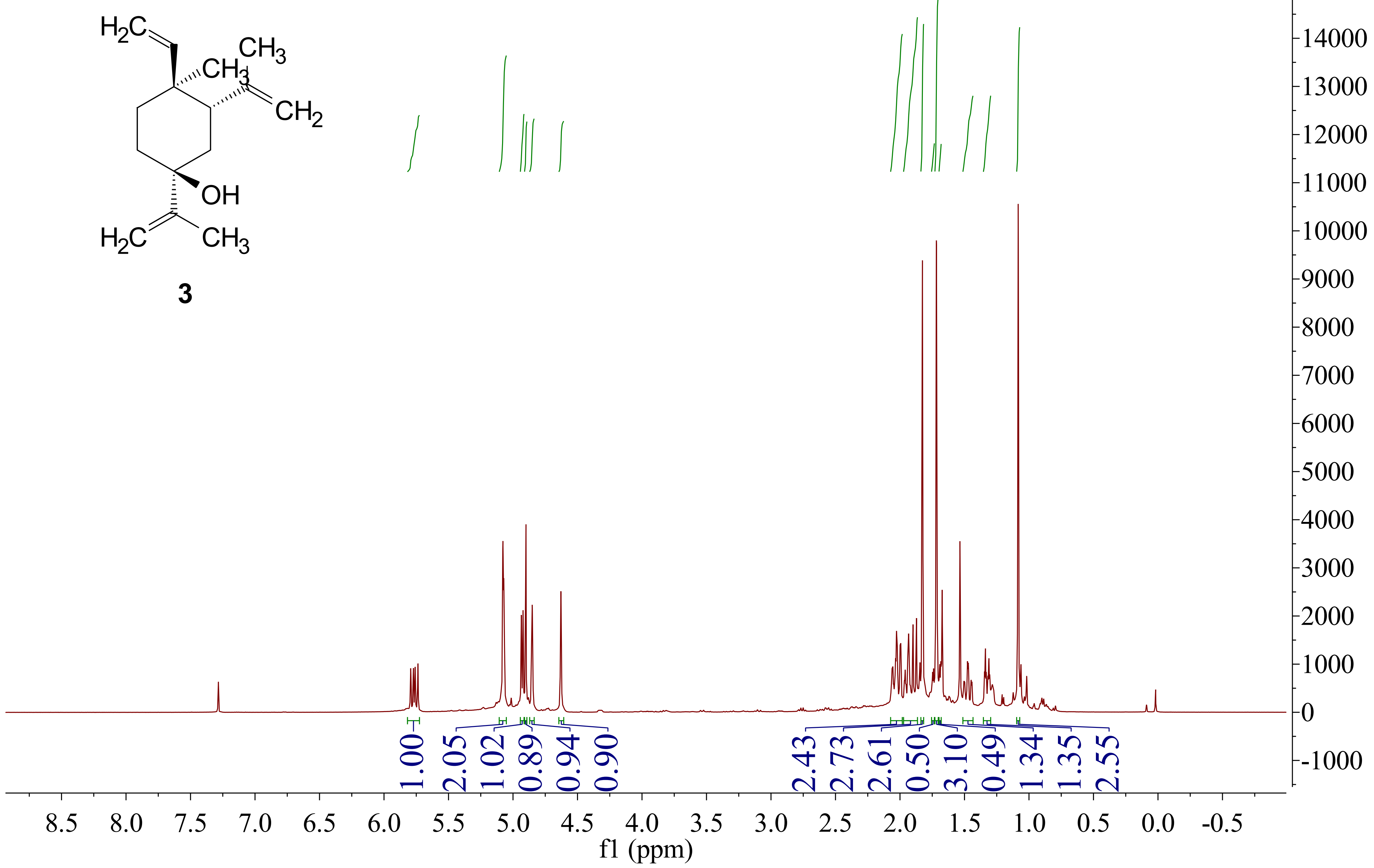

^{13}C NMR (126 MHz, Chloroform-*d*) data of **3**

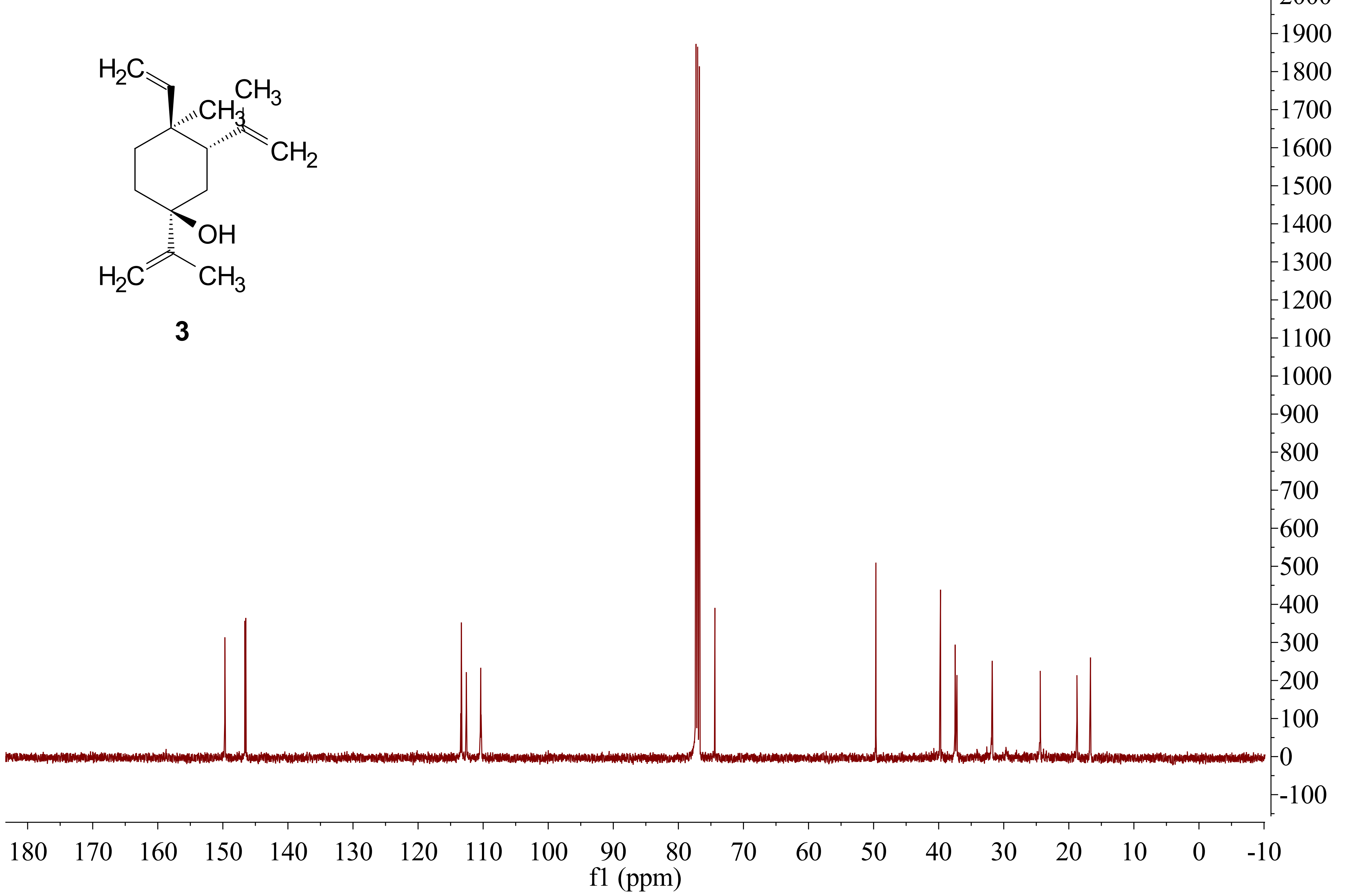

^1H-^1H COSY (Chloroform-*d*) data of **3**

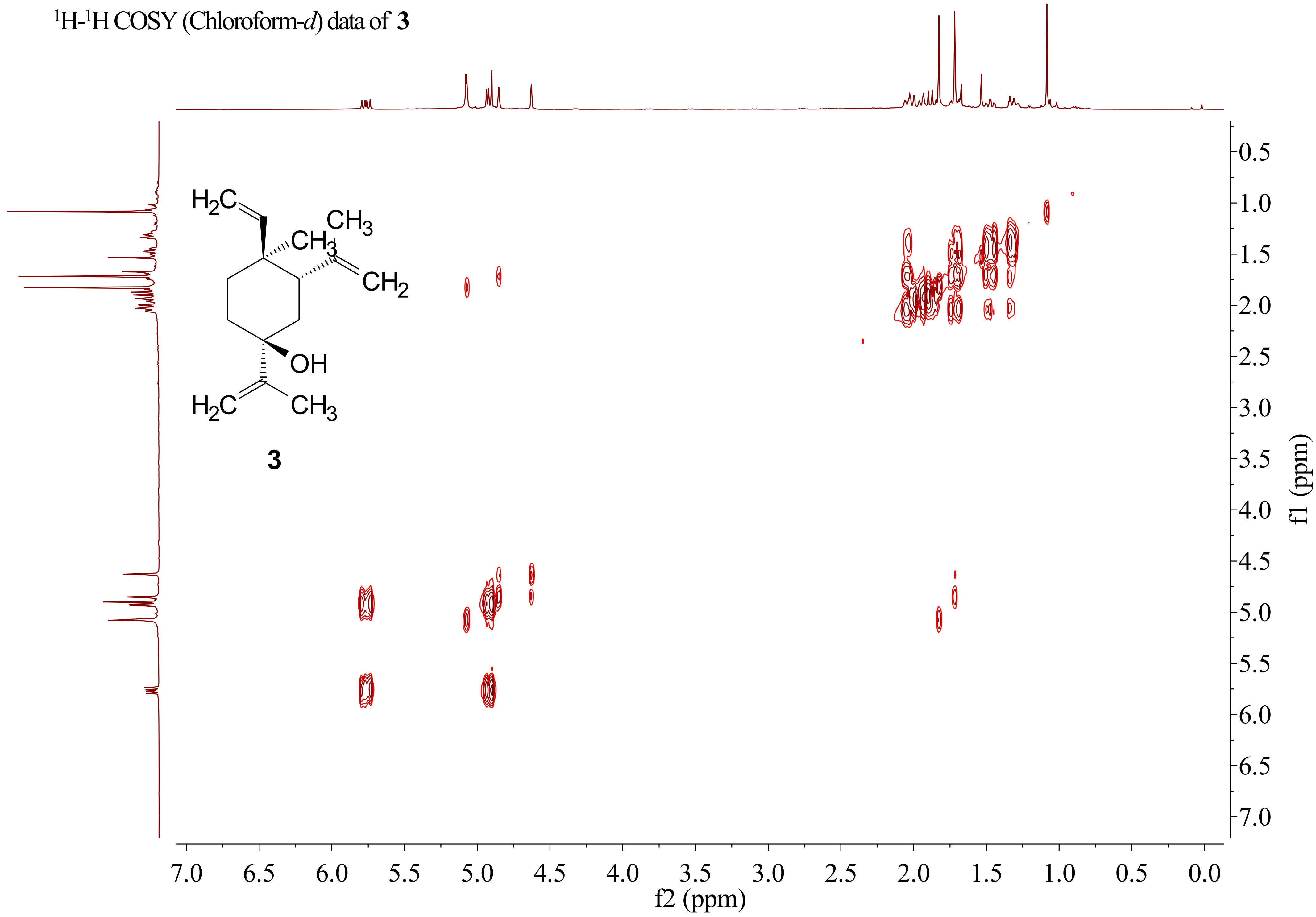

^{1}H-^{1}H COSY (Chloroform-*d*) data of **3**

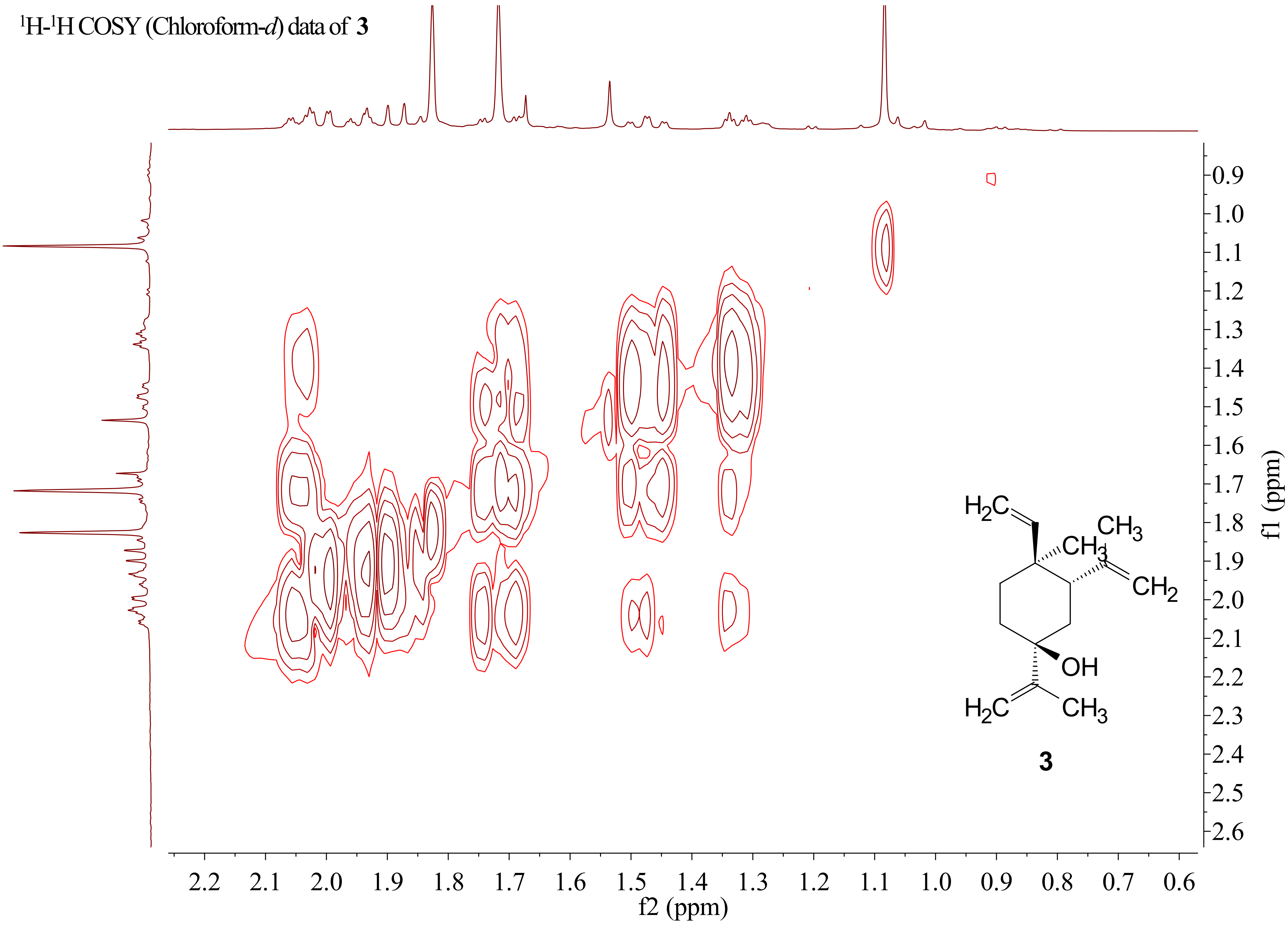

3

HMBC (Chloroform-*d*) data of **3**

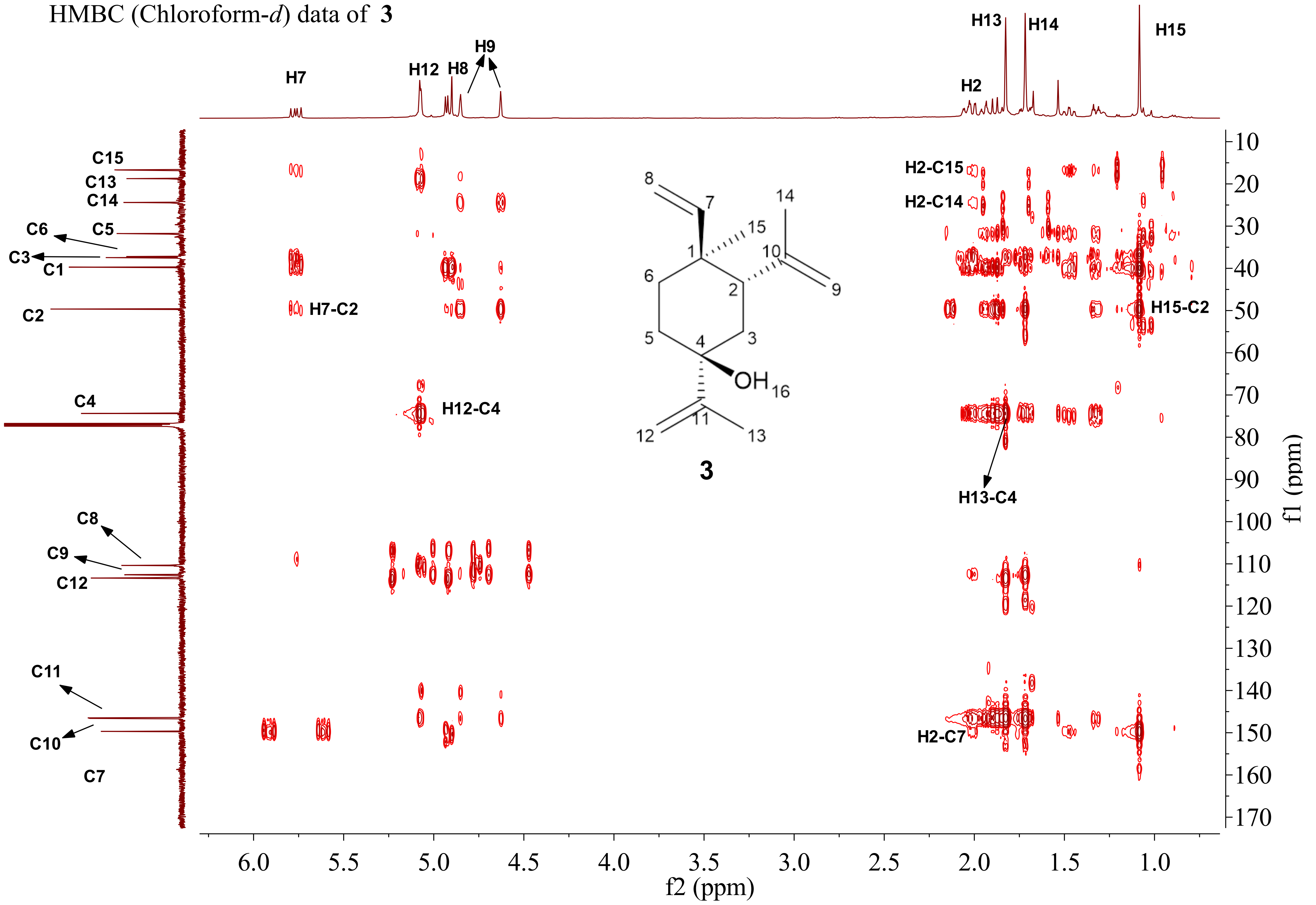

HSQC (Chloroform-*d*) data of **3**

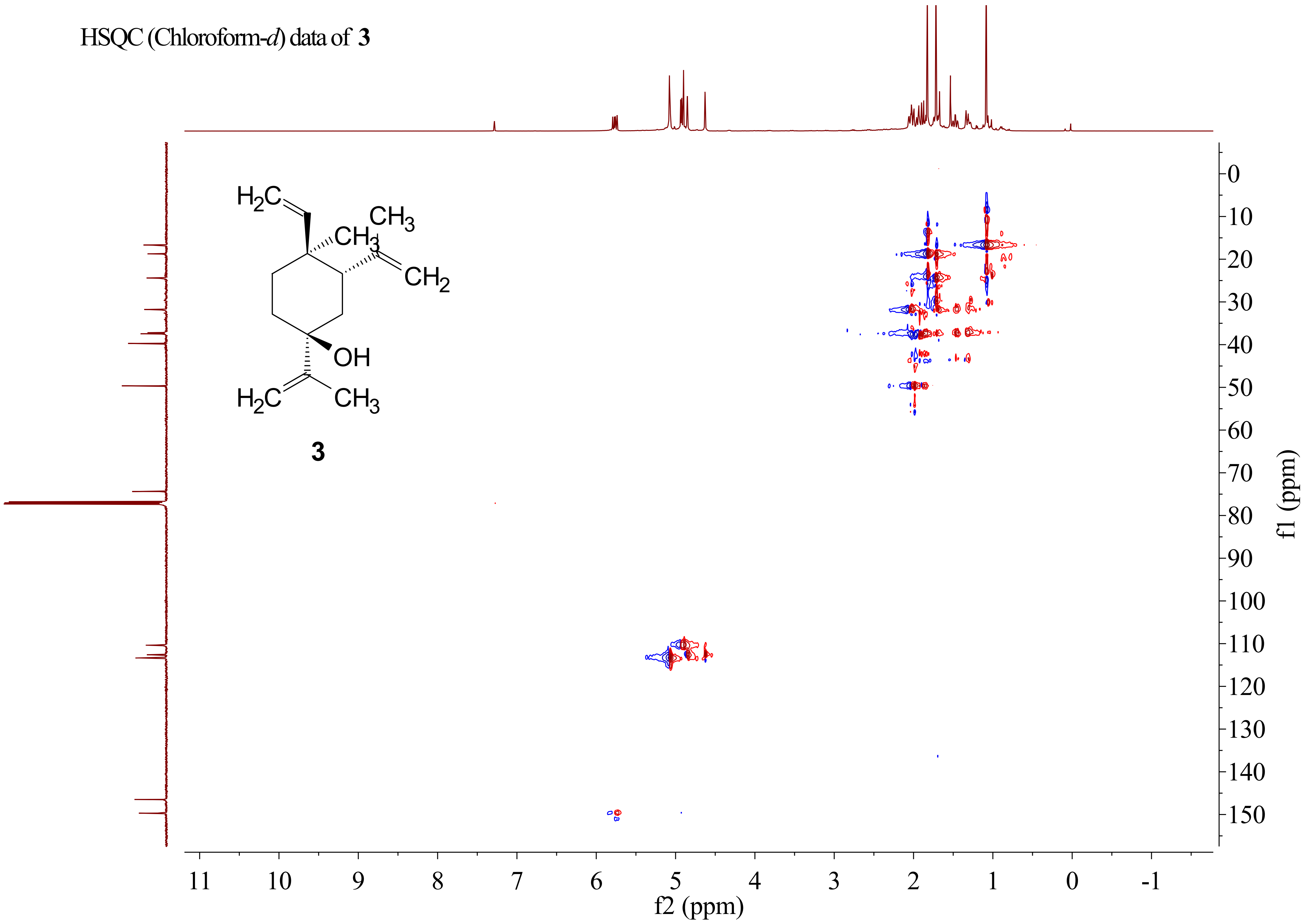

HSQC (Chloroform-*d*) data of **3**

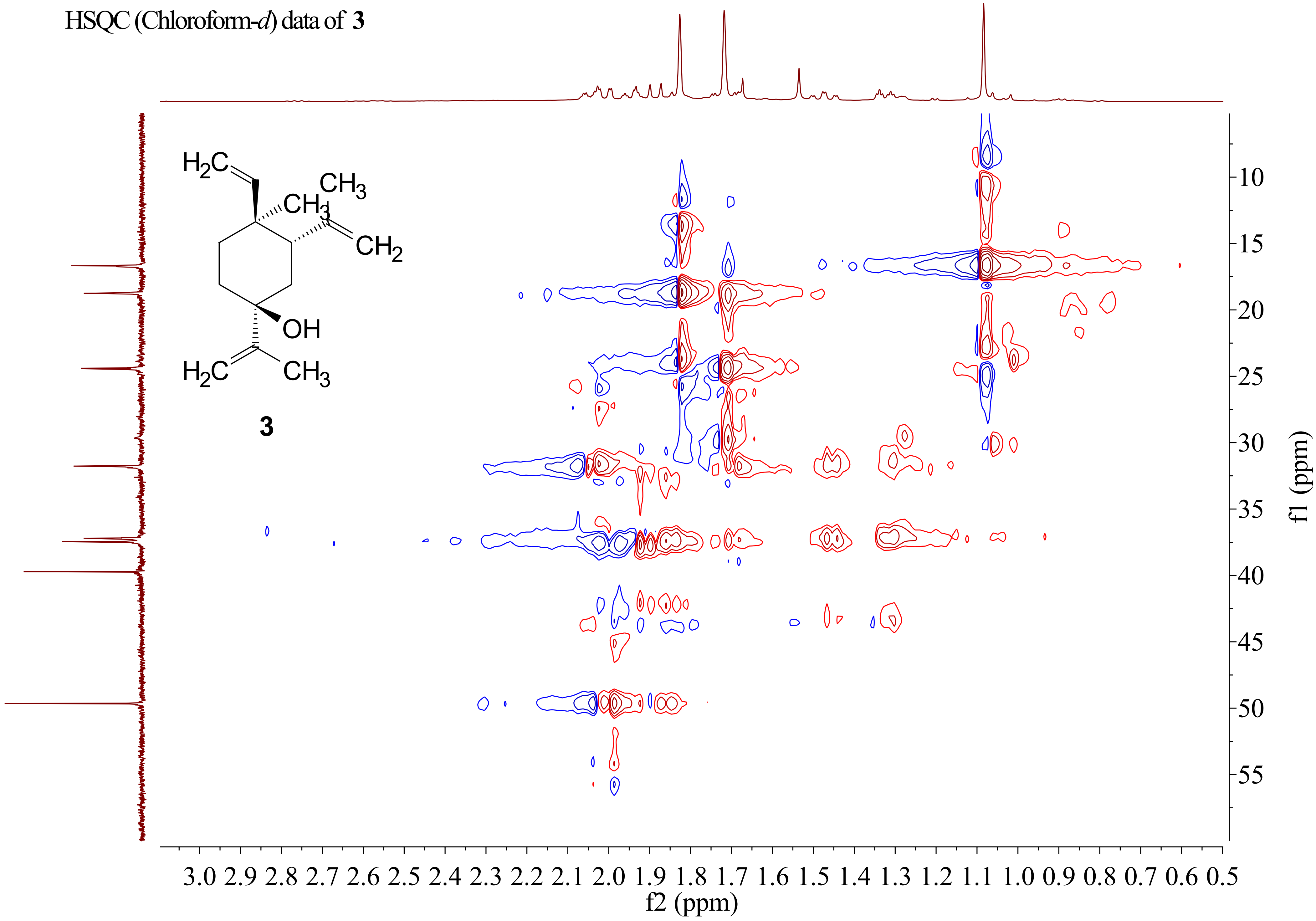

^{1}H NMR (400 MHz, Chloroform-*d)* data of 8

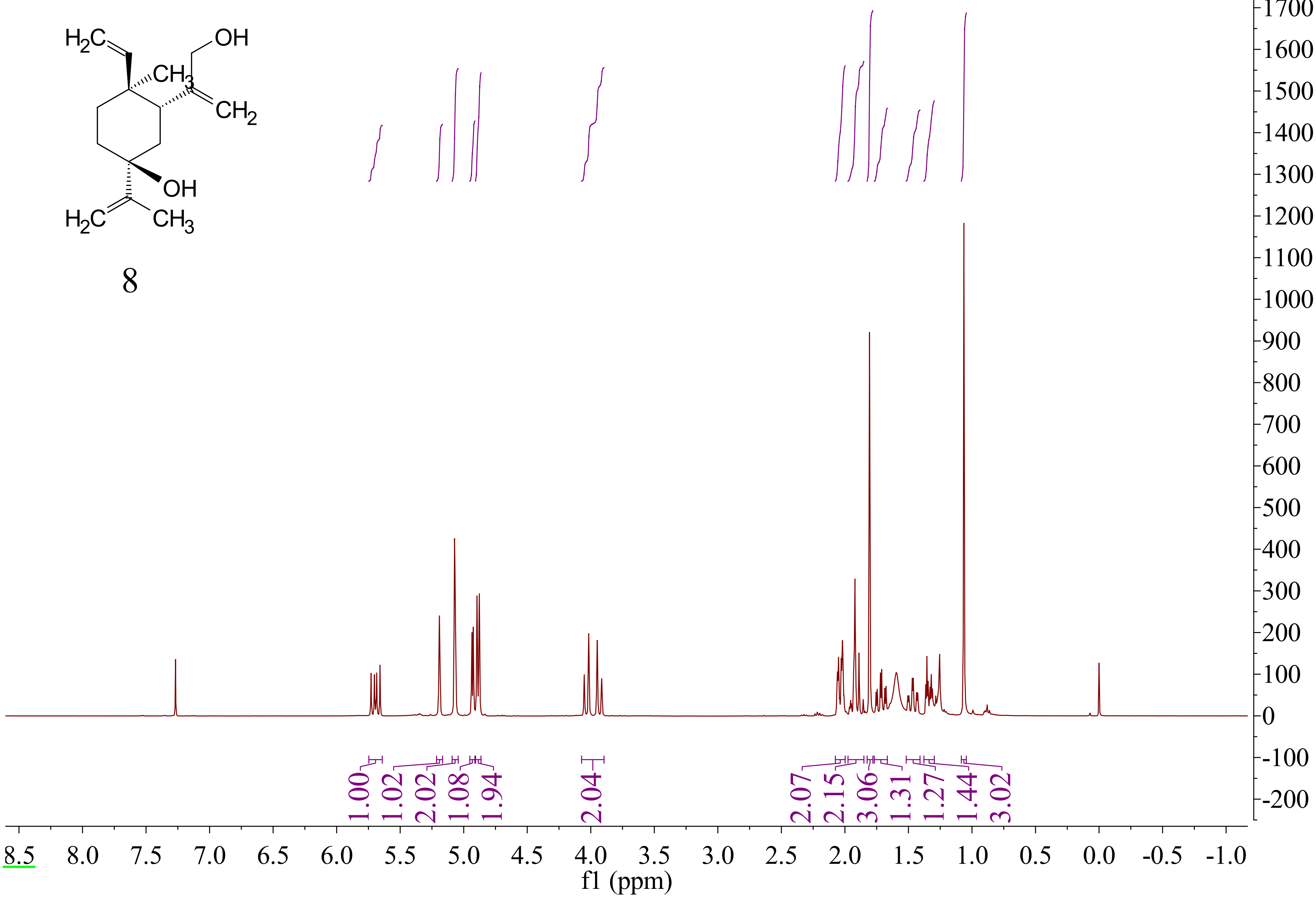

^{13}C NMR (126 MHz, Chloroform-*d*) data of **8**

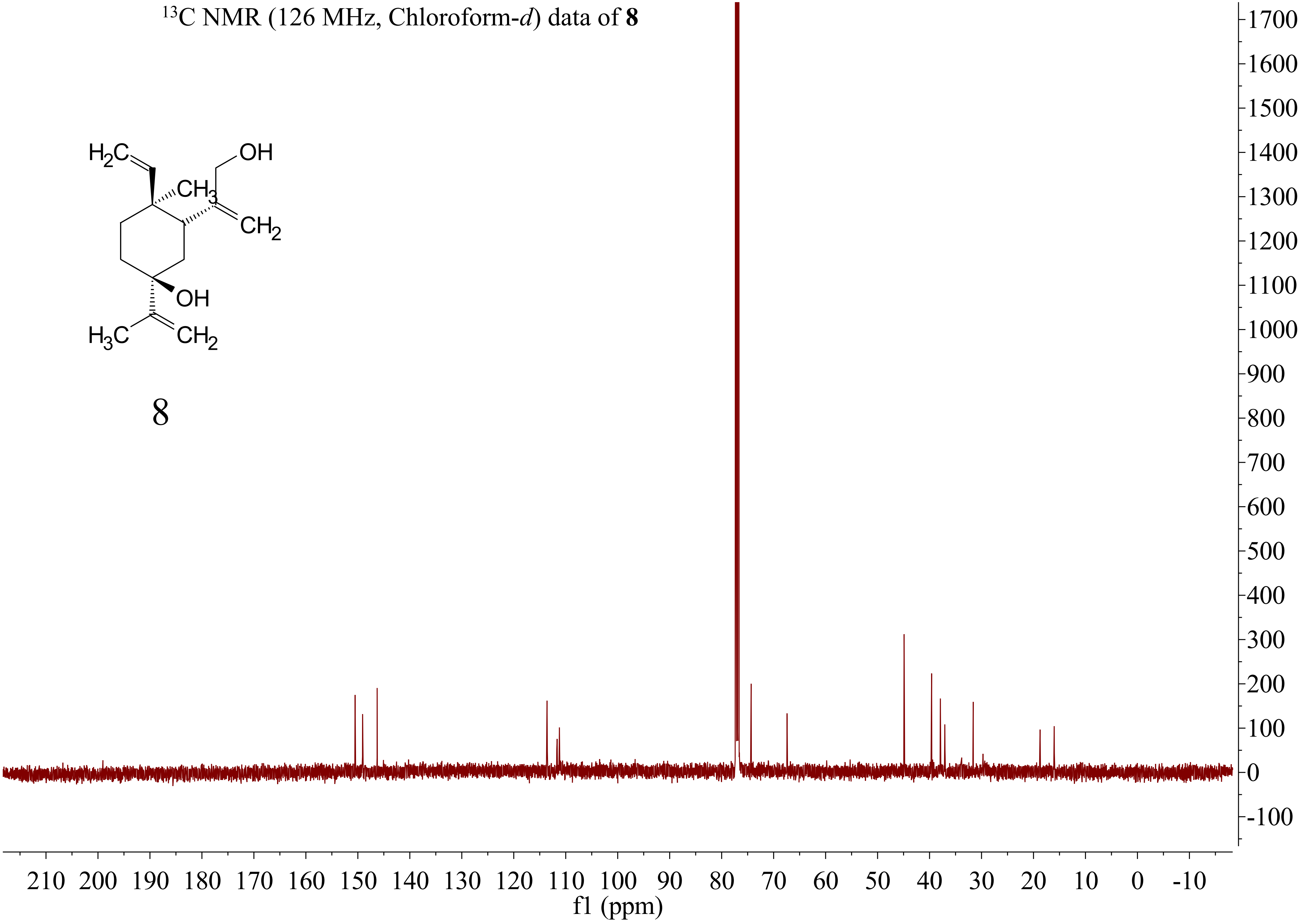

^{1}H-^{1}H COSY (Chloroform-*d*) data of **8**

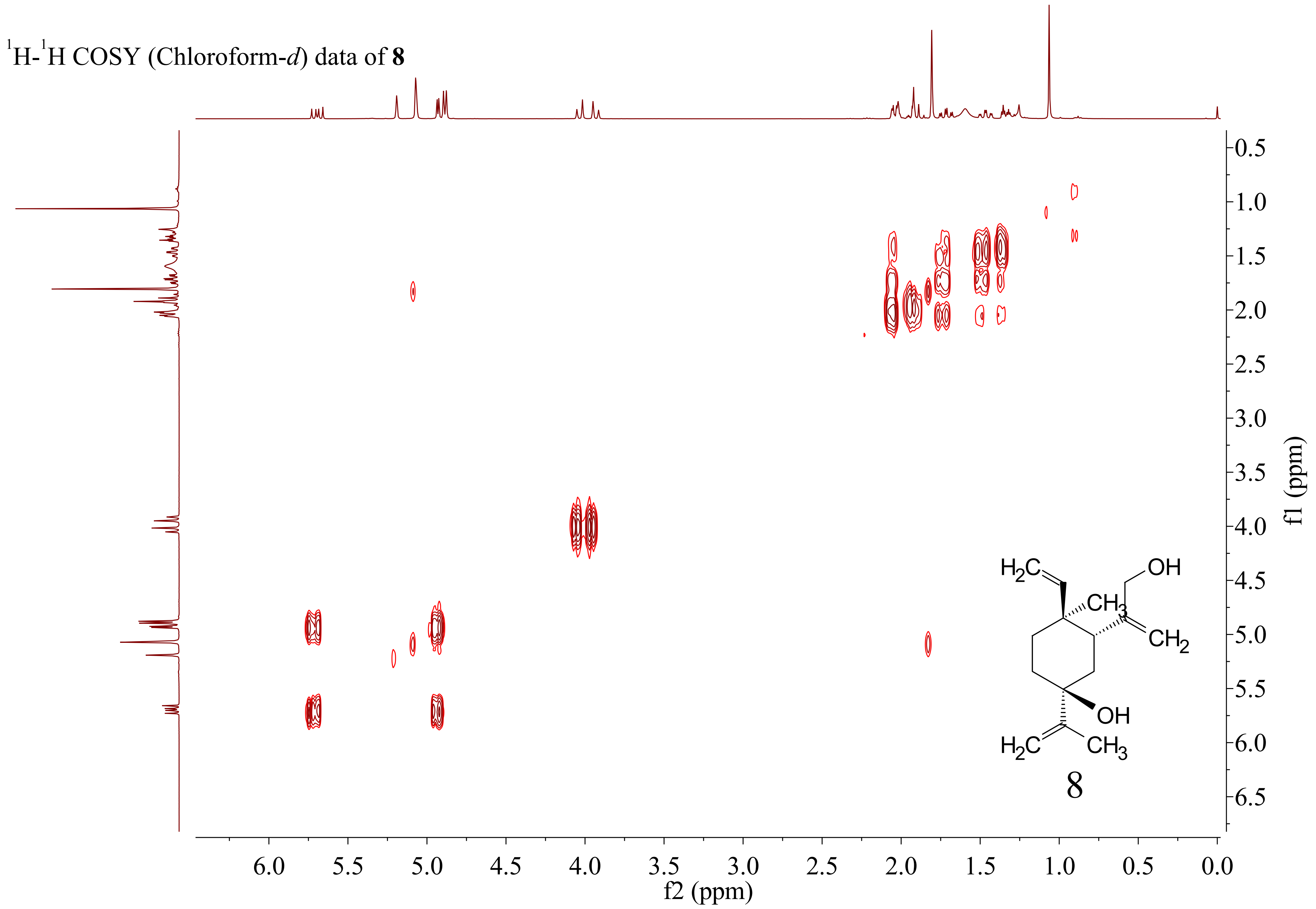

HSQC (Chloroform-*d*) data of **8**

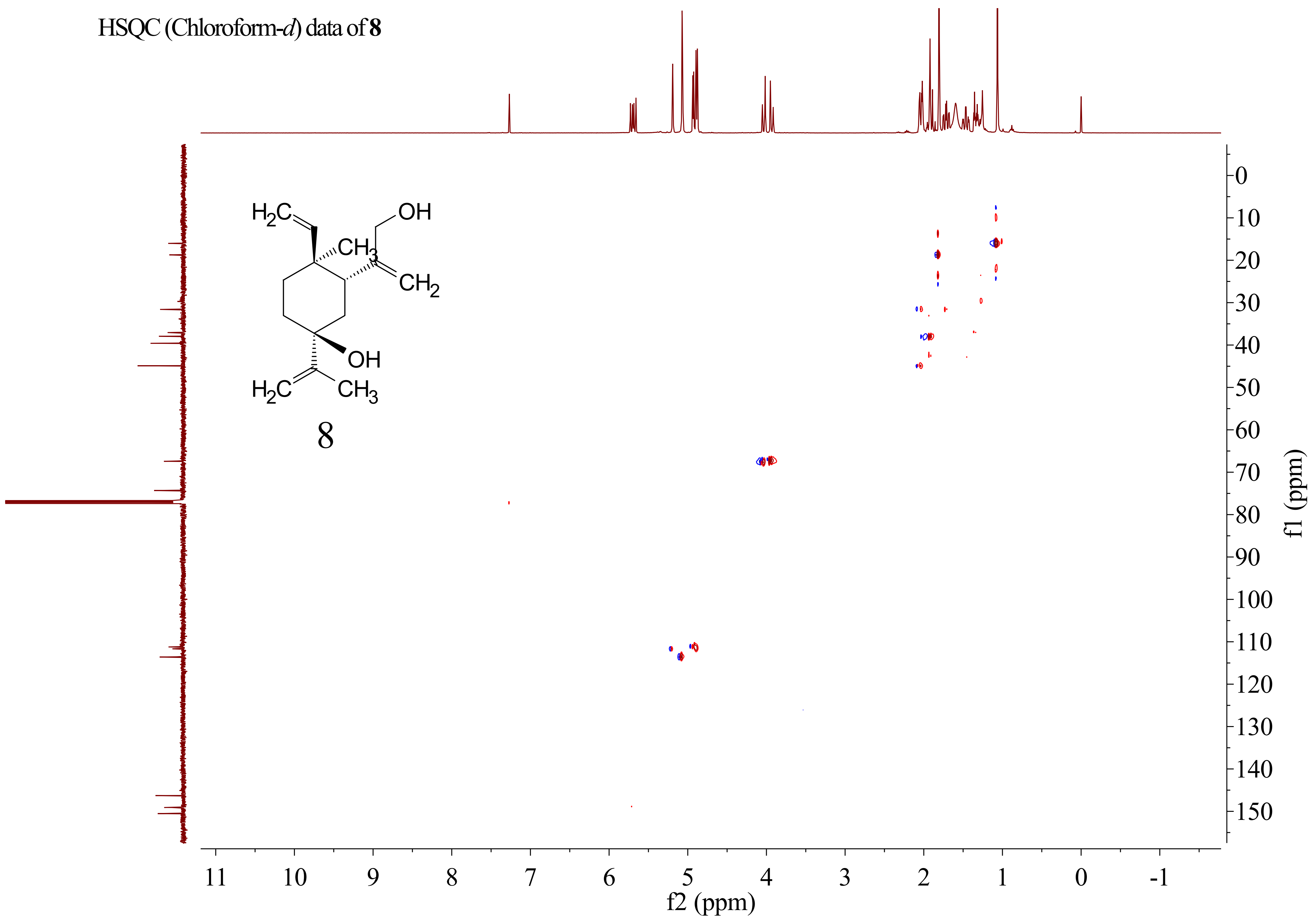

HMBC (Chloroform-*d*) data of **8**

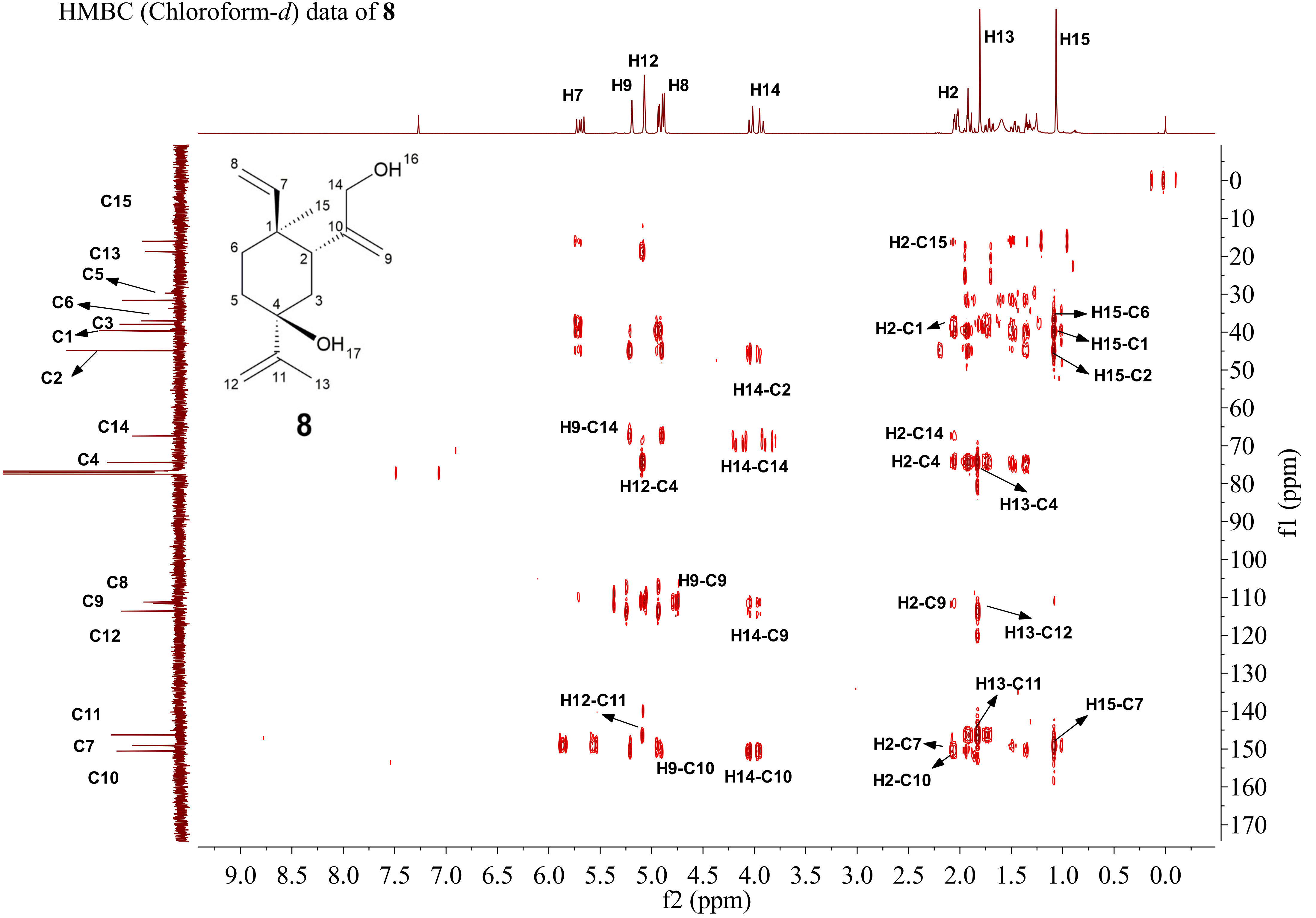

NOESY (Chloroform-*d*) data of **8**

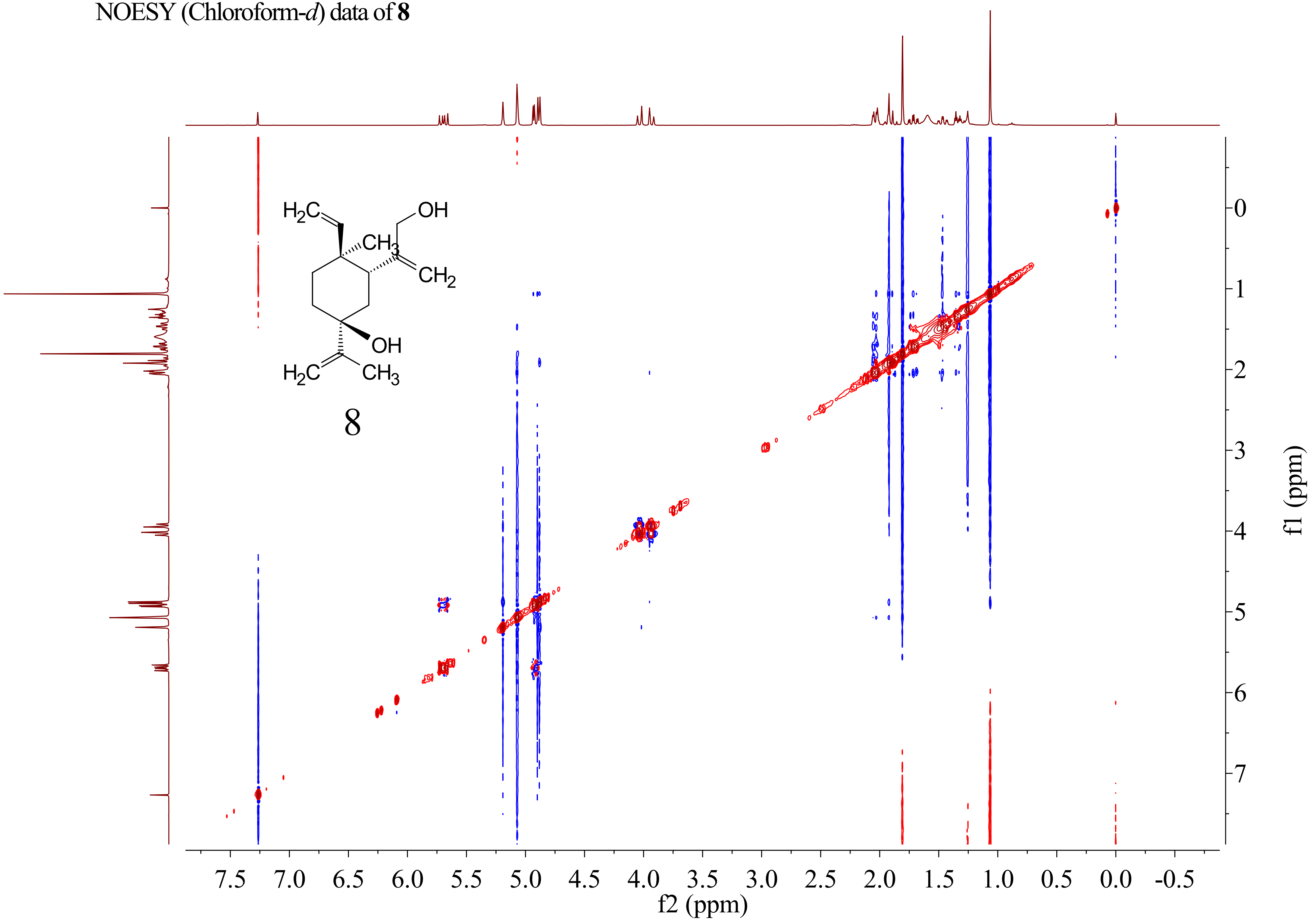

^{1}H NMR (400 MHz, Chloroform-*d*) data of **9**

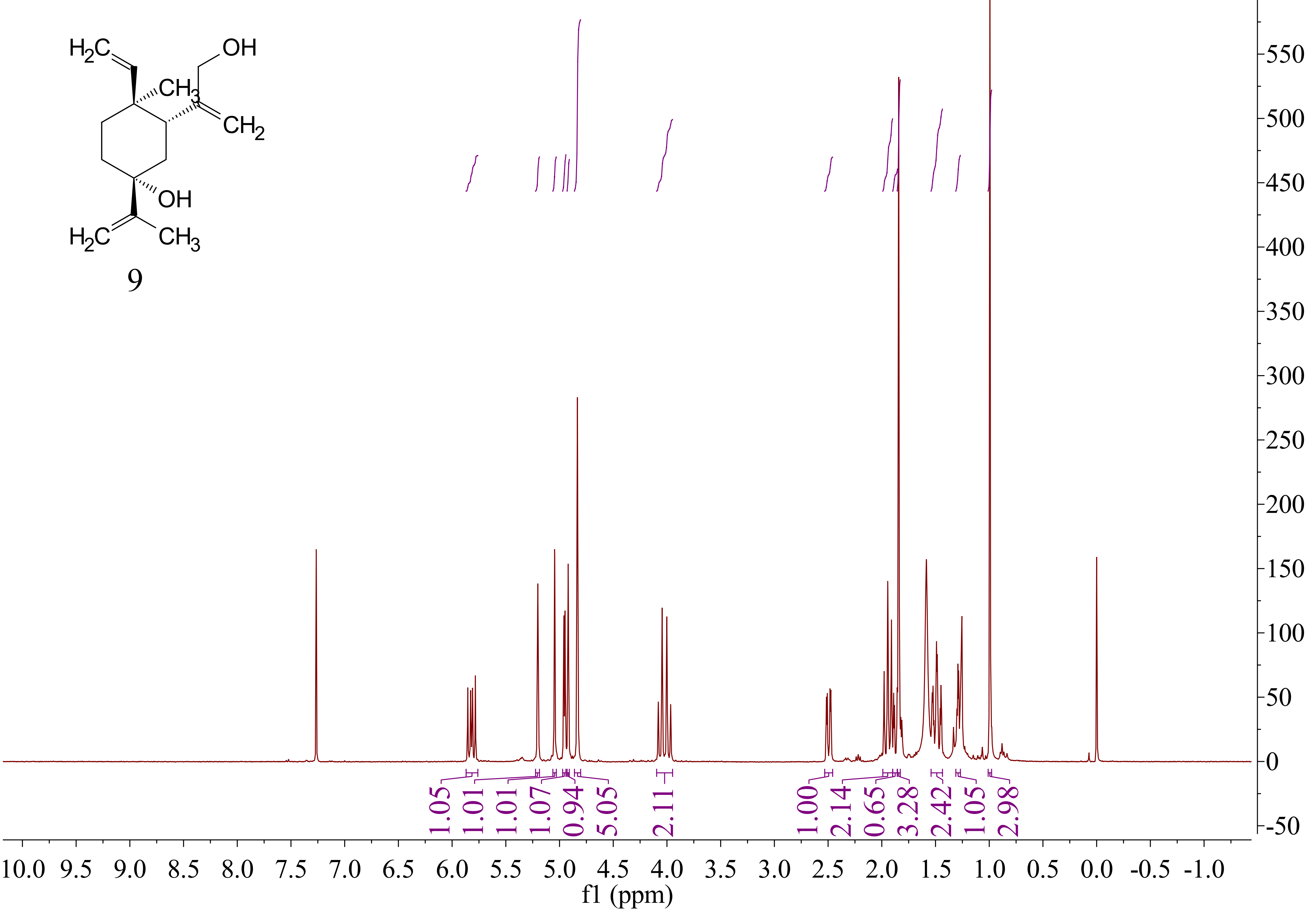

^{13}C NMR (126 MHz, Chloroform-*d*) data of **9**

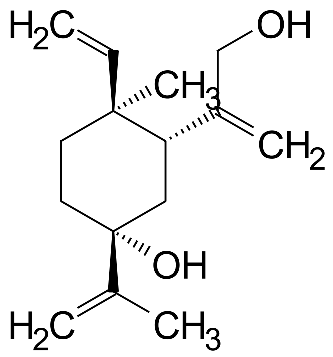

9

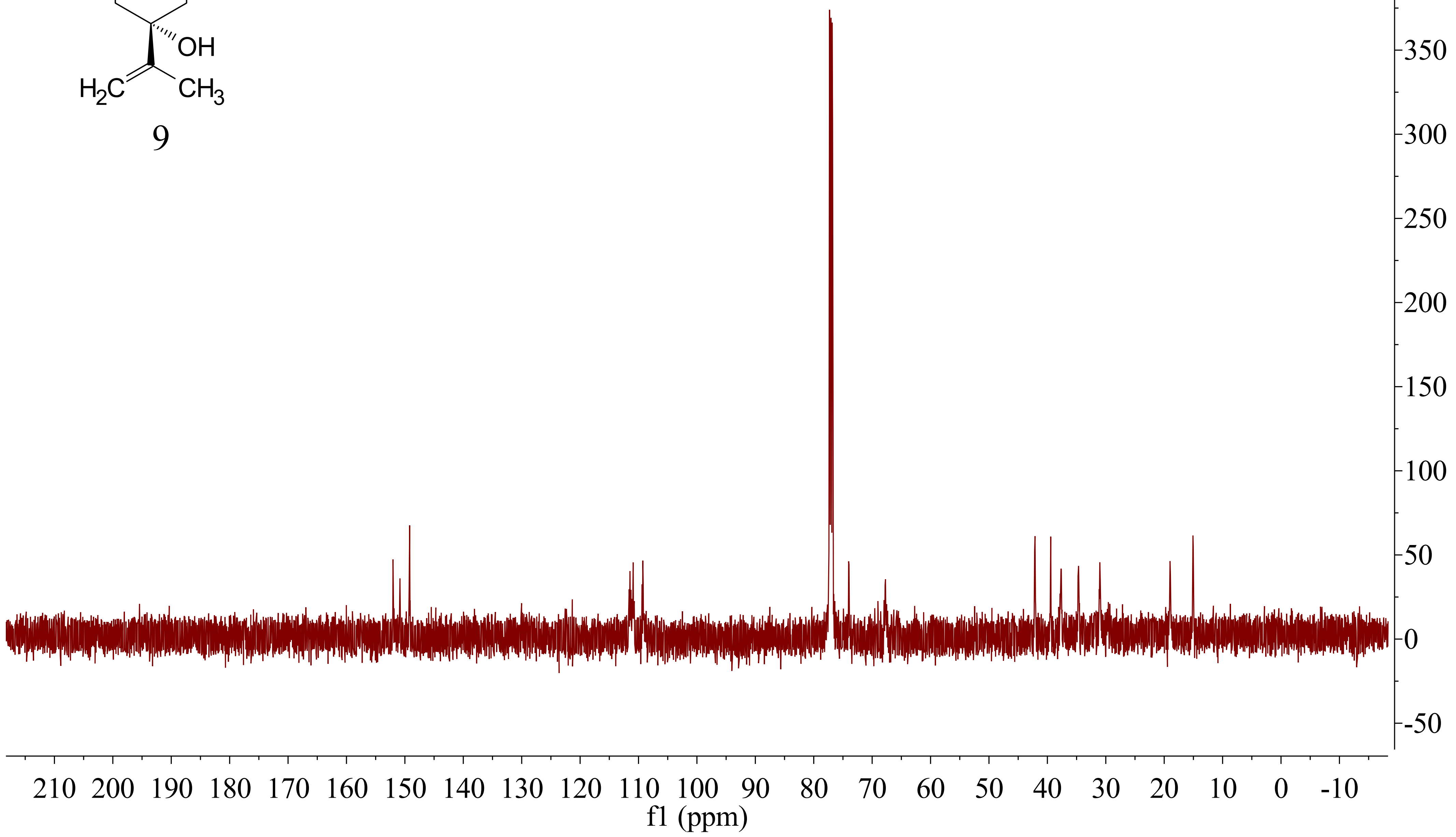

^{1}H NMR (500 MHz, DMSO-d_6) data of **9**

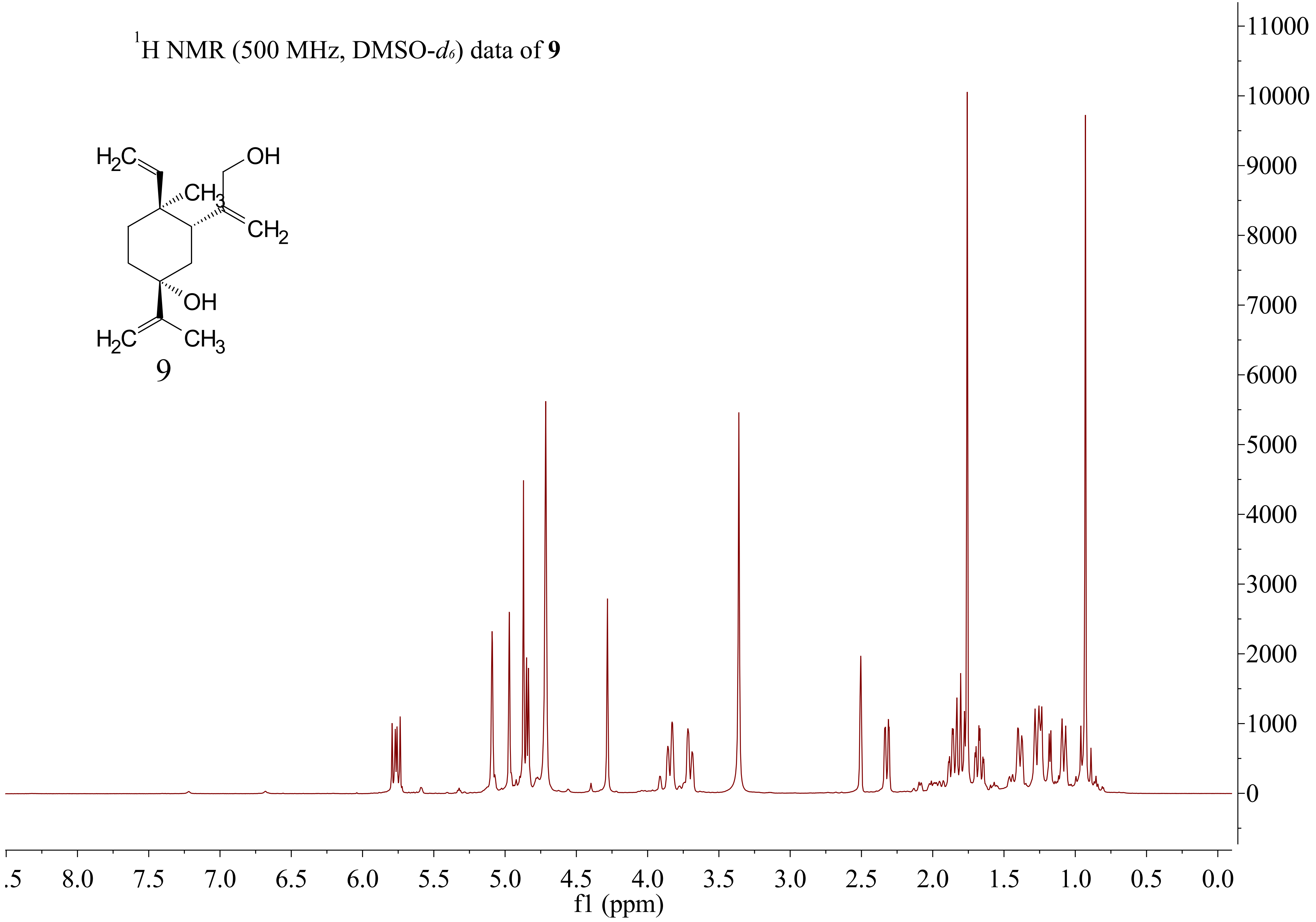

^{1}H-^{1}H COSY (Chloroform-*d*) data of **9**

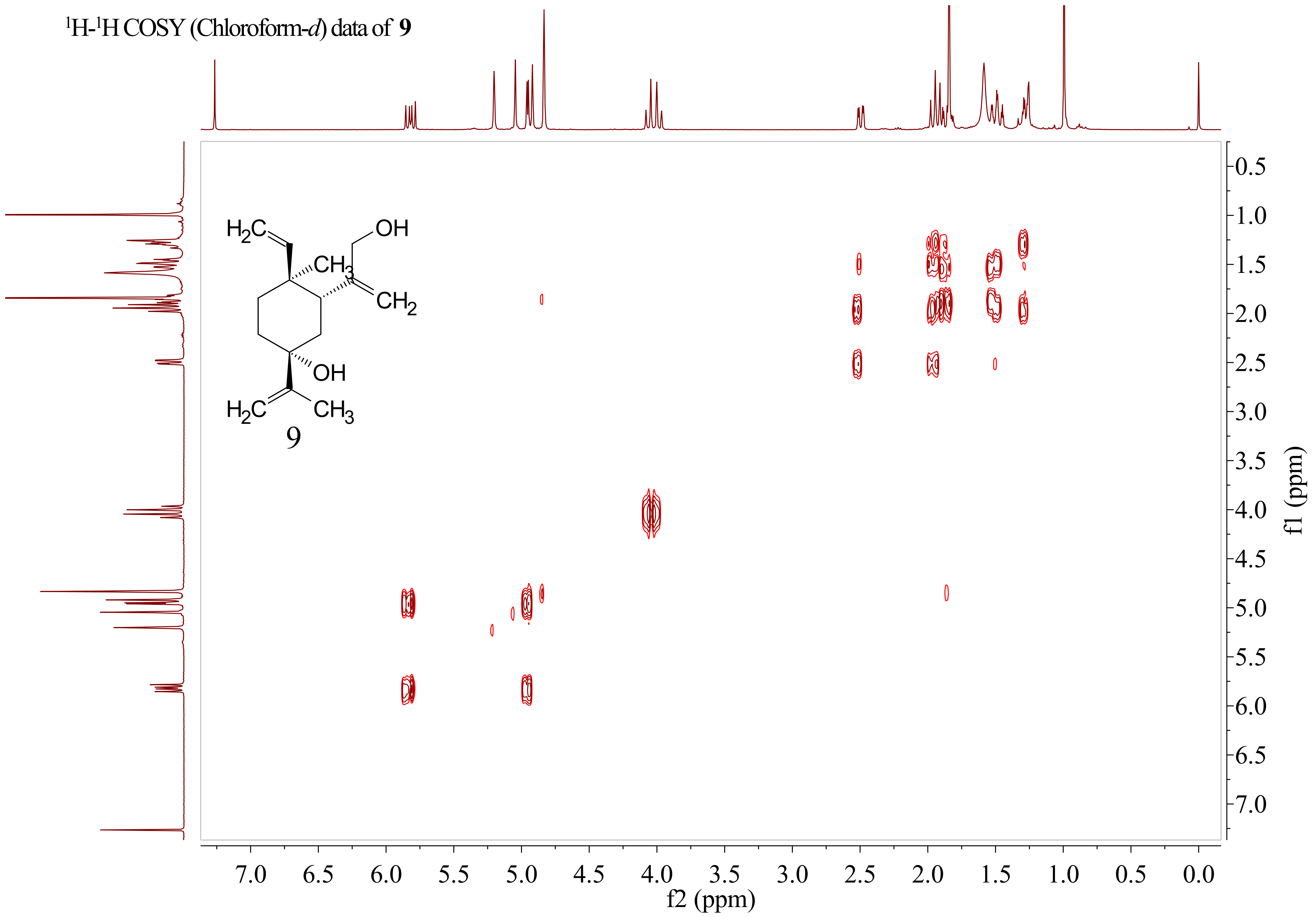

^1H-^1H COSY (Chloroform-*d*) data of **9**

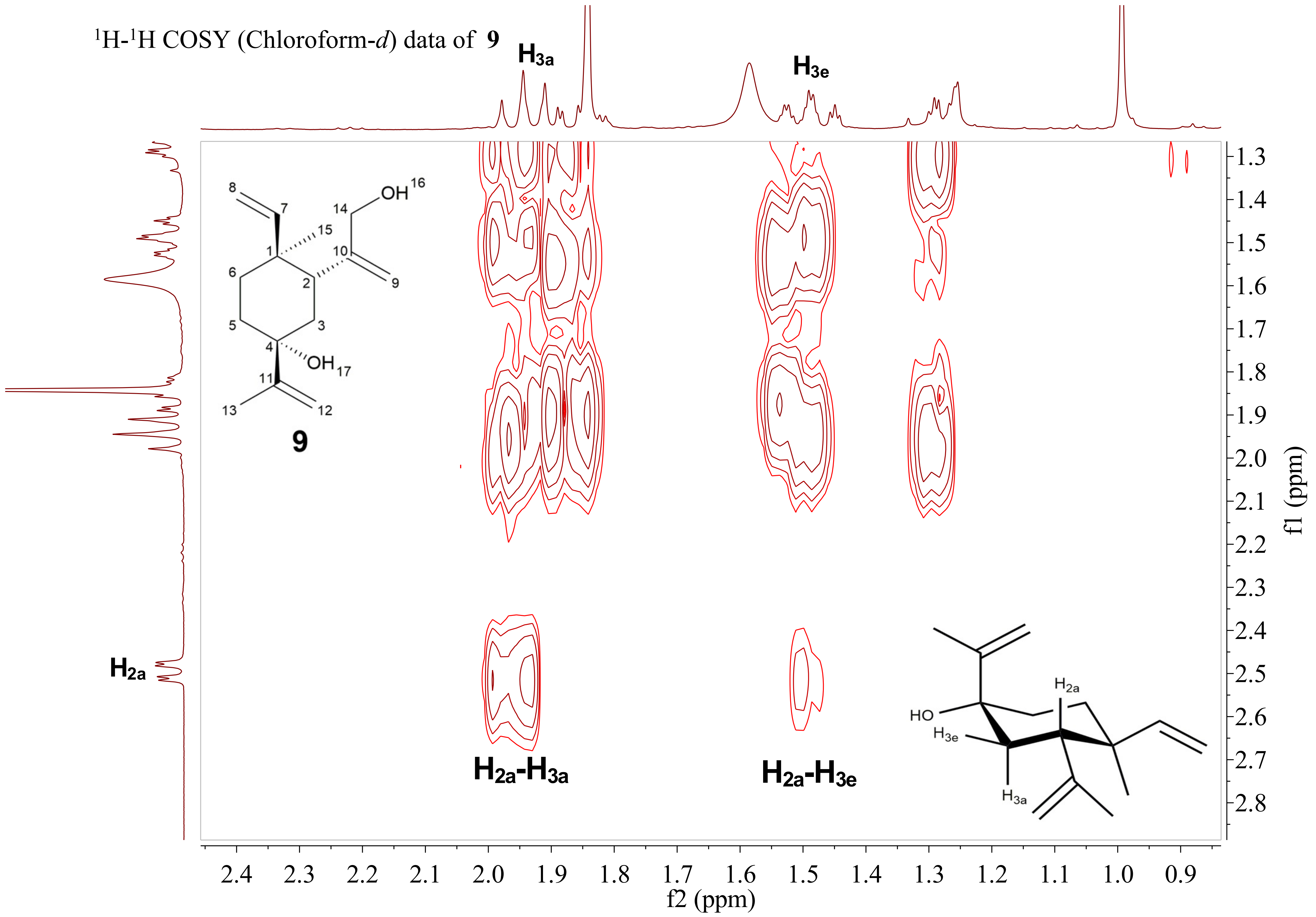

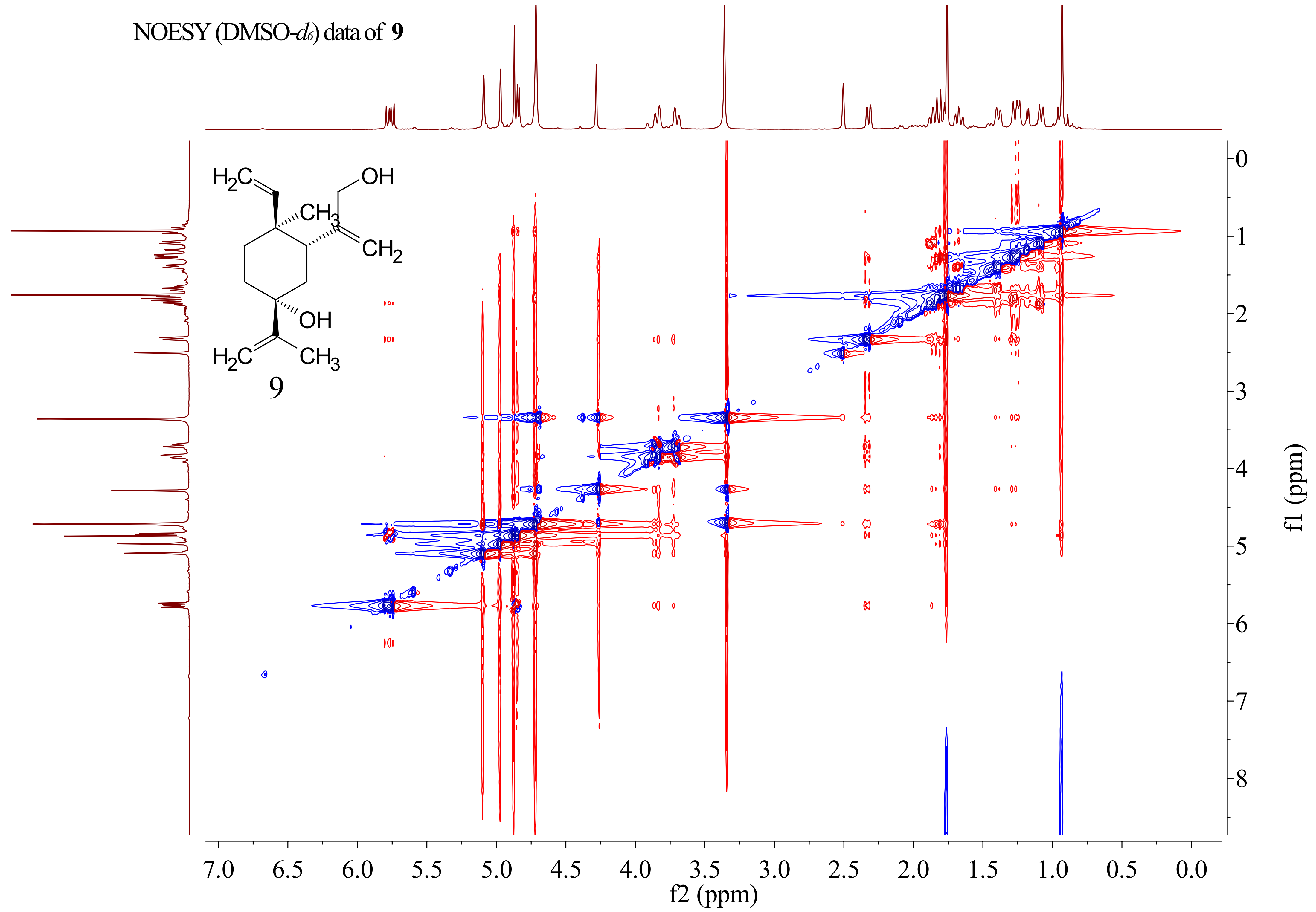
NOESY (DMSO-d_6) data of **9**
H_2C
OH
CH_3
CH_2
OH
H_2C
CH_3
9
f2 (ppm)
f1 (ppm)

NOESY (DMSO-d_6) data of **9**

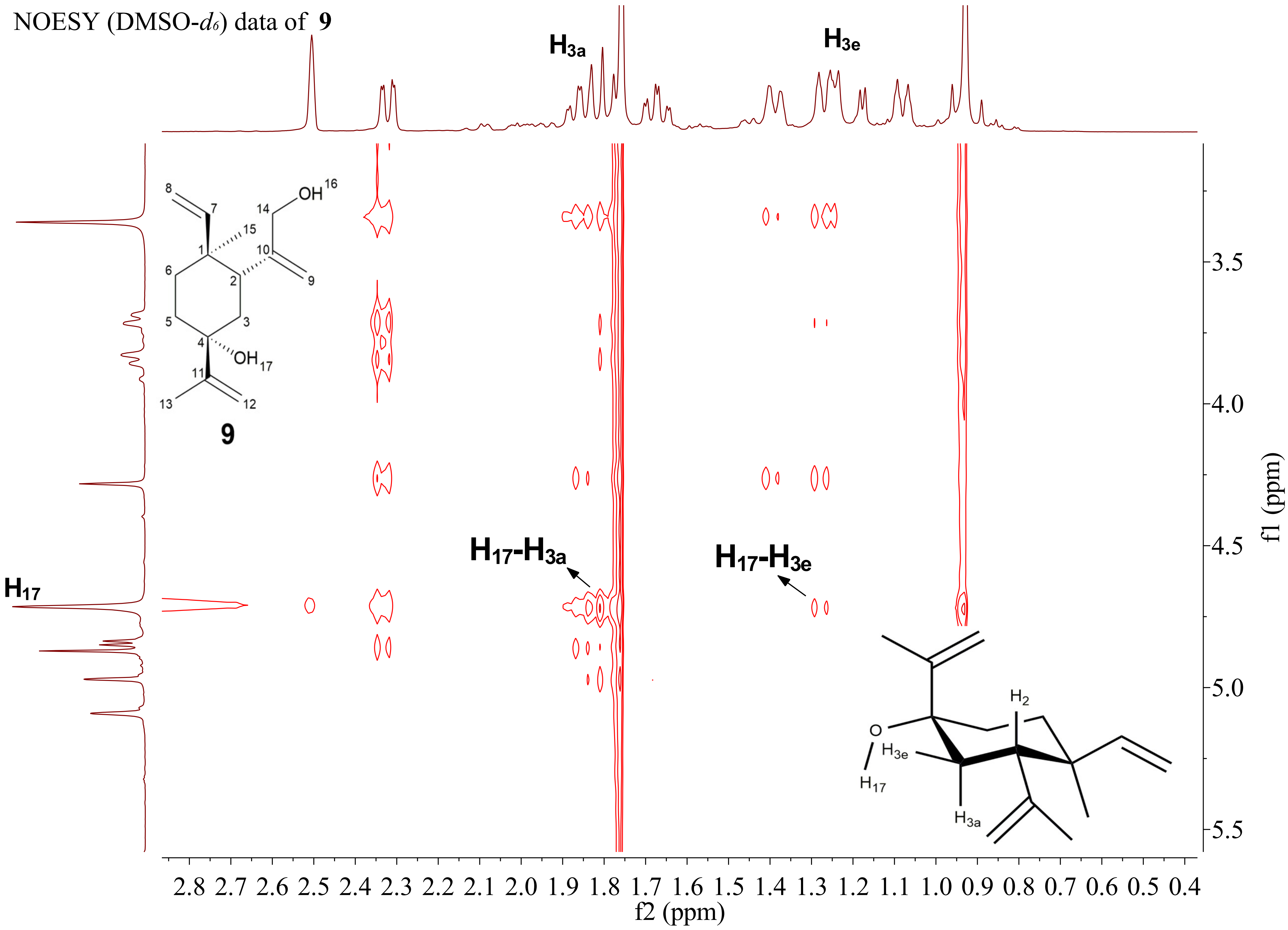

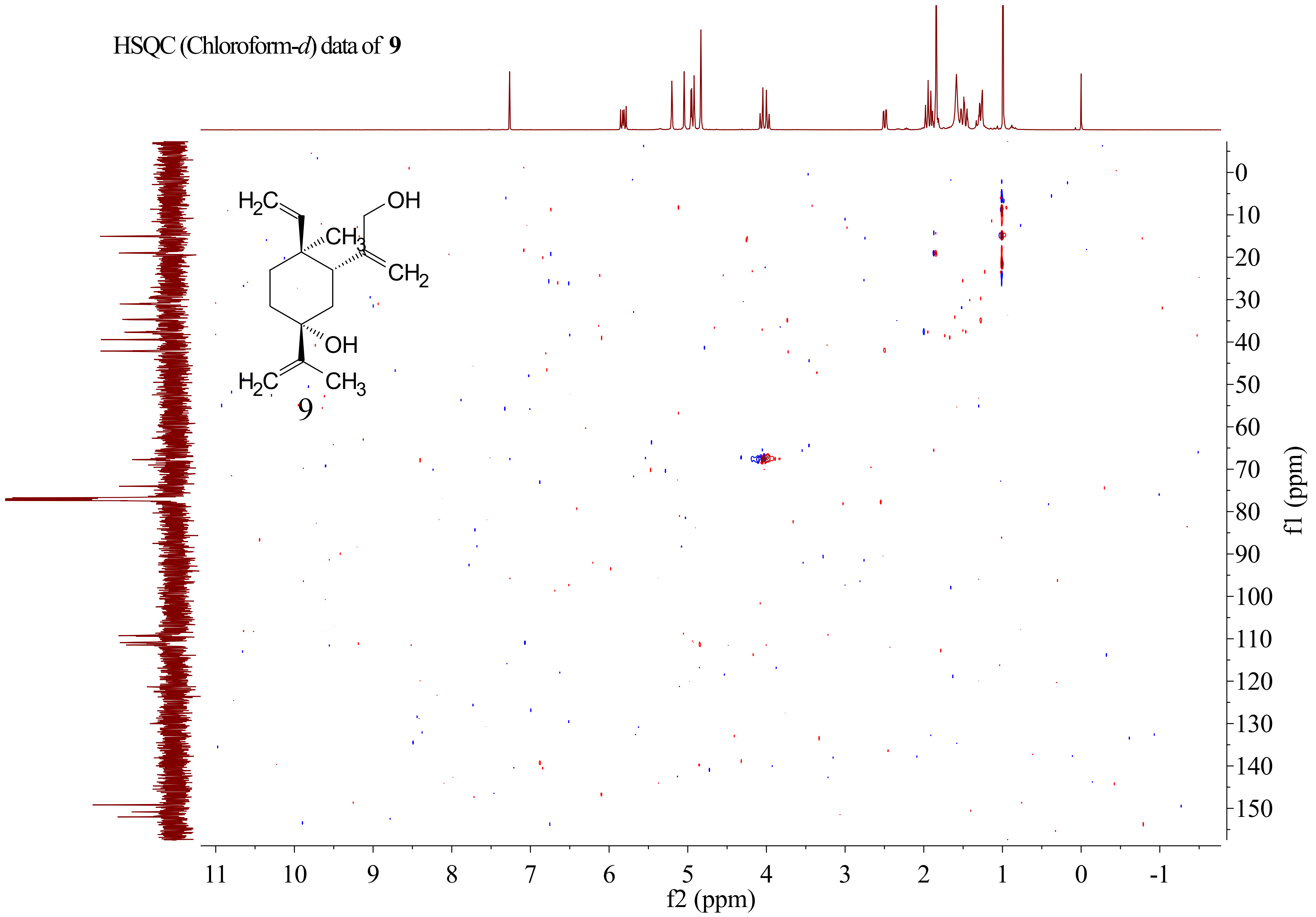

HSQC (Chloroform-d) data of 9
H2C
OH
CH3
CH2
OH
H2C
CH3
9
f1 (ppm)
f2 (ppm)

HMBC (Chloroform-*d*) data of **9**

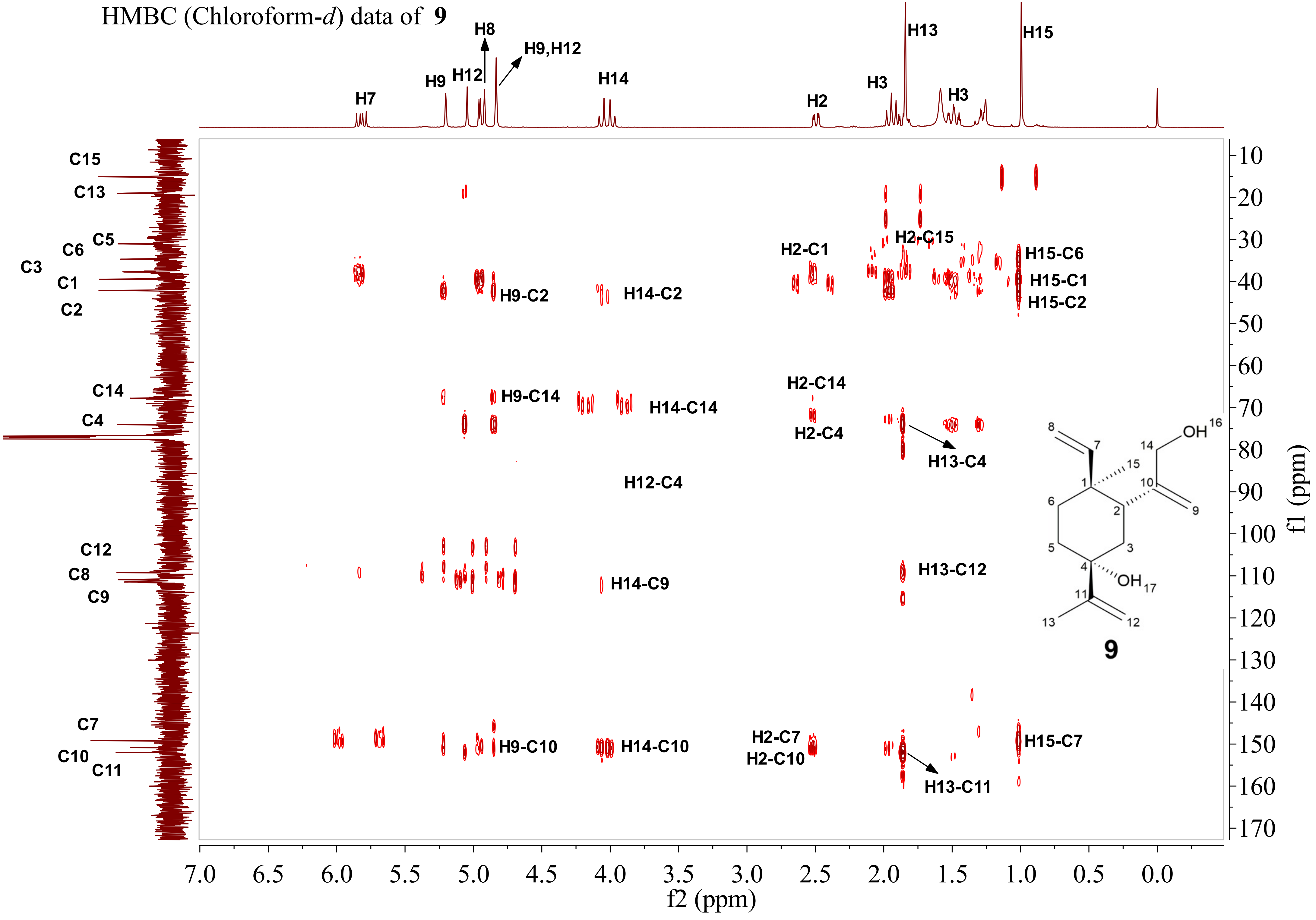

Supplement: Supporting Information [file rsos200038supp1.pdf]
